# Ovarian stimulation with letrozole in nulliparous young women with relapsing early-stage serous borderline ovarian tumors

**DOI:** 10.1016/j.gore.2024.101531

**Published:** 2024-10-15

**Authors:** Valeria Lombardi Fäh, Federico Del Vento, S. Intidhar Labidi-Galy, Manuela Undurraga

**Affiliations:** a Division of Gynecology, Department of Pediatrics and Gynecology, Hôpitaux Universitaires de Genève, Genève, Switzerland; b Department of Oncology, Hôpitaux Universitaires de Genève, Genève, Switzerland; c Faculty of Medicine, Department of Medicine and Center of Translational Research in Onco-Hematology, University of Geneva, Swiss Cancer Center Leman, Genève, Switzerland

**Keywords:** Ovarian stimulation with letrozole in serous borderline tumors

## Abstract

Fertility preservation (FP) is an important aspect of the treatment of young women diagnosed with serous borderline ovarian tumors (SBOT), with fertility sparing surgery recommended when possible. Concurrent treatment with aromatase inhibitor letrozole during ovarian stimulation (OS) could be used in women with hormone-sensitive breast cancer, but very little is known in gynecological tumors. Here, we report the cases of 2 young nulliparous women with early stage SBOT who underwent successful OS with letrozole. Patient 1 is 22-years old, FIGO IIB. She had a bilateral ovarian recurrence 5 months after the first surgery. She underwent OS with letrozole (four oocytes were collected and vitrified), followed by cytoreduction. The patient is in complete remission since 2 years. Patient 2 is 27-years old, FIGO IC3, treated by right adnexectomy. Ten months after surgery, she was in complete remission. OS with letrozole was performed and 4 oocytes were retrieved, resulting in 2 blastocysts that were cryopreserved. She had a successful pregnancy after in-vitro fertilization. She underwent a delivery via C-Section for obstetrical reasons that revealed a macroscopic suspicious lesion on the left ovary. Cystectomy was performed during C-section, confirming tumor recurrence. She underwent a second pregnancy uneventfully. During the second C-section, a partial cystectomy and multiples peritoneal biopsies were performed revealing tumor recurrence limited to the left ovary. She underwent left adnexectomy two months after C-Section without any recurrence. In conclusion, our case report described successful oocytes cryopreservation, without changes in the appearance of ovarian cysts, in nulliparous women with early-stage SBOT who underwent OS with simultaneous administration of letrozole.

## Highlights

• Successful fertility preservation after ovarian stimulation with letrozole in young women with relapsing early-stage serous borderline ovarian tumors.
• Both patients had successful oocyte retrieval despite tumor *in situ*.
• No changes observed in ovarian cysts after ovarian stimulation with letrozole.
• One patient completed two successful pregnancies.

## 1 Introduction

Serous borderline ovarian tumors (SBOT) arise mainly among women of reproductive age who have not completed childbearing. Fertility preservation (FP) is an important aspect of their comprehensive care, particularly if they are young and nulliparous (Morice et al., 2024). Fertility sparing surgery (FSS) is recommended whenever possible. It involves repeated surgical procedures that could compromise fertility such as unilateral or bilateral ovarian cystectomy and/or adnexectomy and/or hysterectomy. Scientific societies recommend fertility consultation before debulking surgery (Oktay et al., 2018). Traditional FP methods, including oocyte or embryo cryopreservation, typically requires ovarian stimulation (OS). However, these conventional OS protocols can lead to elevated estrogen levels (Lambertini et al., 2017), raising concerns on their oncological safety, particularly in patients with hormone-sensitive gynecological tumors. This is particularly true for SBOT for which an increased risk of incidence was reported in women exposed to fertility treatments (Buonomo and Peccatori, 2020).

In the last decade, clinical protocols incorporating aromatase inhibitors have been developed to enable OS without increasing estrogen levels, therefore allowing women with hormone-sensitive tumors to undergo FP (Oktay et al., 2005). A *meta*-analysis in 2020 provides reassuring insights, indicating that the incorporation of letrozole into OS does not adversely impact the yield of mature oocytes and is correlated with a significant reduction of estradiol levels peak (Bonardi et al., 2020).

Data on OS with letrozole in women with hormone-sensitive tumors were mainly reported in breast cancer. Importantly, FP does not elevate the risk of cancer recurrence or mortality in women with hormone-sensitive breast cancer (Goldrat et al., 2022).

Currently, very little is known on the use of OS in the context of early-stage SBOT. Here, we report the case of two young nulliparous patients of reproductive age diagnosed with early-stage SBOT who underwent successful OS with gonadotrophins and concomitant administration of letrozole, without deterioration of the oncological stage.

## 2 Case series

### 2.1 Patient

A nulliparous 22-year-old woman was operated in a private clinic for bilateral ovarian cysts with elevated CA-125 levels (115 kU/L). She underwent laparoscopy with peritoneal cytology, bilateral cystectomy and multiples peritoneal biopsies that established the diagnosis of FIGO IIB SBOT. One month after diagnosis, a fertility consultation was conducted, and the patient expressed a desire to preserve her fertility but did not follow-up with her decision.

Five months later, the patient was referred to Geneva University Hospitals due to recurrence, identified via ultrasound and magnetic resonance imaging (MRI), revealing a 2.5 cm cyst in the right ovary and a 3.2 cm cyst in the left ovary, while CA-125 levels were normal (26 kU/l). She was offered to perform OS for oocytes cryopreservation. The OS was performed using Letrozole 2.5 mg 2 pills/day. The patient received 3000 U of recombinant FSH (GonalF R), Ganirelix 0.25 mg (Orgalutran R) was administered daily after day 6 of stimulation and for ovulation double-triggering Triptorelin 0.2 mg (Decapeptyl R 0.1 mg/ml) and 250 microg of recombinant human choriogonadotropin (Ovitrelle R) were used. Four mature oocytes were retrieved and vitrified.

**Fig. 1:**
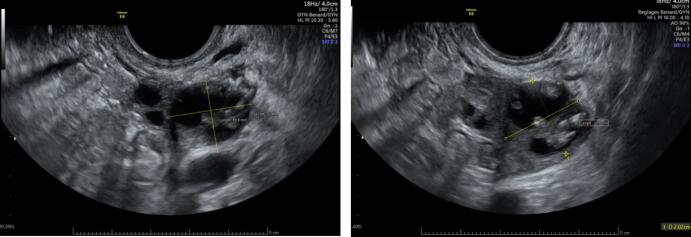
**Stability of SBOT in patient 1 after ovarian stimulation (OS). **Left ovary with a cyst of 19x22 mm before OS (A) and 20x21 mm two weeks after OS (B).

Three weeks after cryopreservation, pelvic ultrasound showed a stability of the size of bilateral cysts, measuring 3 cm in the right ovary and 2 cm in the left ovary **(**Fig. 1**)**.

The patient underwent a cytoreduction with partial right and left oophorectomy, right salpingectomy, appendicectomy, omentectomy and multiples biopsies, confirming a stage FIGO IIB. After cytoreduction, the left ovary returned to function with a resumption of menstrual cycles. AMH levels dropped from 10 pmol/ml to 0.4 pmol/ml. Reduction of the ovarian reserve was confirmed by pelvic ultrasound that showed an antral-follicle-count of 2. Since the patient insisted in her commitment to fertility preservation procedures, further treatments were scheduled, although the expected result was discussed to be lower compared to the first OS. Hence, the patients subsequently performed two unsuccessful cycles of ovarian mild-stimulation with concomitant 10 mg/day letrozole administration that did not result in retrieval of any mature oocyte. The first one with 900 U of human gonadotrophins (Merional R), daily injection after day 6 of stimulation of 0.25 mg Ganirelix (Orgalutran R) and ovulation double triggering with Triptorelin 0.2 mg (Decapeptyl R 0.1 mg/ml) and 250 microg of recombinant human choriogonadotropin (Ovitrelle R). 2 oocytes were retrieved but none was mature. A last cycle was abandoned for insufficient response after 6 days of stimulation with 900 U of human gonadotrophins (Merional R). The patient is in complete remission 2 years after the last surgery.

### 2.2 Patient

A nulliparous 27-year-old-woman was operated for a right ovarian cyst with elevated CA-125 levels (60 kU/l). She underwent laparoscopy with peritoneal cytology, right adnexectomy and multiples biopsies, leading to the diagnosis of FIGO IC3 SBOT. After surgery, the patient conducted a fertility consultation with her partner and expressed her wish to preserve fertility.

Ten months after surgery, she was in complete remission. An OS under 5 mg daily administration of Letrozole was performed. An antagonist protocol applying daily injection after day 6 of stimulation of 0.25 mg Ganirelix (Orgalutran R) and 2025 U of human gonadotrophins (Merional) was used. After double-triggering with Triptorelin 0.2 mg (Decapeptyl R 0.1 mg/ml) and 250 microg of recombinant human choriogonadotropin (Ovitrelle R), 4 mature oocytes were retrieved, resulting in 2 blastocysts that were cryopreserved. One of these embryos was transferred during a natural modified cycle, where ovulation was triggered at day 16 with 250 microg of recombinant human choriogonadotropin (Ovitrelle R).

The patient had a pregnancy and a delivery via C-Section at term for obstetrical reasons. During the C-section surgery, cystectomy of the left ovary was performed due to the presence of a suspicious macroscopic lesion. Pathology analysis confirmed tumor recurrence.

Five months following the recurrence diagnosis, a 8 mm cyst with vegetations was identified in the left ovary during follow-up ultrasound and confirmed by MRI. The patient underwent two consecutive OS cycles. In the first attempt, 2700 U of recombinant follicle stimulating hormone (rFSH) and recombinant luteinizing hormone (rLH) (Pergoveris, R) were used, daily administration of Ganirelix started at day 6, double-trigger of ovulation was obtained with Triptorelin 0.2 mg (Decapeptyl R 0.1 mg/ml) and 250 microg of recombinant human choriogonadotropin (Ovitrelle R). No oocyte was retrieved after this OS. For the second OS, 2700 U of recombinant FSH (GonalF R) and 1350 U of human gonadotrophins (Merional R) were administered, Ganirelix was prescribed starting at day 6 of stimulation, double-trigger of ovulation was achieved with Triptorelin 0.2 mg (Decapeptyl R 0.1 mg/ml) and 250 microg of recombinant human choriogonadotropin (Ovitrelle R). Three matures oocytes were obtained, resulting in two two-days embryos. One embryo was cultured *in vitro* up to the stage of blastocyte and frozen as a fertility preservation procedure. The second embryo was transferred after 2 days of *in vitro* culture and resulted in pregnancy.

**Fig. 2:**
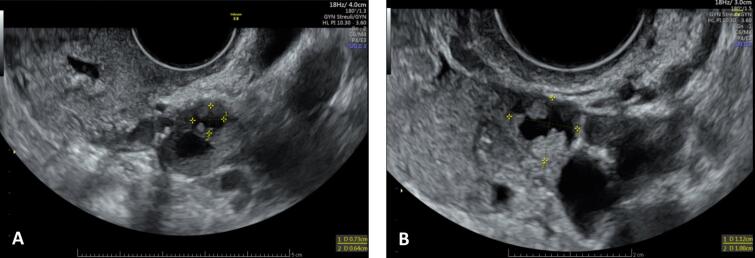
**Stability of SBOT in patient 2 after ovarian stimulation (OS).** Left ovary with a cyst of 10x8.2 mm before OS (A) and 11x10 mm six weeks after OS (B).

At 8 weeks of gestation, pelvic ultrasound revealed the persistence of the cyst with vegetations in the left ovary, measuring 11 mm (Fig. 2).

Close monitoring continued throughout the pregnancy, which was uneventful. C-Section was performed at term. During C-Section, a left partial ovariectomy was performed with peritoneal cytology and multiple peritoneal biopsies. It confirmed a recurrence of SBOT confined to the remaining ovary measuring 12 mm with no other recurrence site and negative cytology. Laparoscopic staging with left adnexectomy was performed two month after the C-Section, and no tumor recurrence was observed. Since the patient still has 2 viable embryos and there are no signs of extra-ovarian disease, a hysterectomy has not been scheduled.

## 3 Discussion

The management of borderline ovarian tumors (BOT) has evolved due to a better understanding of their biological behavior. FSS is now safely offered to patients with early-stage SBOT who have not completed childbearing, supported by evidence that higher recurrence rates do not affect overall survival (Morice et al., 2024, Plett et al., 2020). Recent data suggests FSS should also be considered for advanced stage SBOT, despite the elevated recurrence rates, as it offers reproductive success without impacting overall survival (Falcone et al., 2022). The ESGO-ESHRE-ESGE guidelines now recommend fertility-sparing management for BOT at any stage (Morice et al., 2024).

Recurrence factors of BOT include incomplete staging, residual disease, and the choice of FSS, especially with bilateral ovarian involvement. Fertility outcomes after FSS are excellent, with first pregnancy rates over 80 %, highlighting its potential for successful fertility preservation (Plett et al., 2020). However, while stage I SBOT recurrences often represent new primary BOTs in the residual ovary, stage II-IV SBOTs carry a higher risk of recurrent invasive low-grade serous carcinoma with peritoneal spreading (Gershenson, 2017).

For gynecological tumors affecting pelvic organs, FP procedures add complexity as the affected organs and tumoral cells may be sensitive to the drugs used. FSS can lead to incomplete staging during FP. Bilateral ovarian involvement risks iatrogenic tumor spillage during egg retrieval. Additionally, OS for FP in the presence of micrometastases in the contralateral ovary could accelerate tumor spread. Thus, balancing reproductive preservation and tumor management in BOT requires a nuanced approach, distinct from breast cancer challenges.

Managing recurrent SBOT and associated fertility challenges requires a multidisciplinary team, including medical oncologists, gyneco-oncologists, radiologists, and onco-fertility specialists. Our series shows that ovarian surgery impacts ovarian reserve, evident from the number of oocytes collected after OS for both patients and should be discussed thoroughly before surgery. The timing of FP procedures must be carefully assessed based on the clinical situation, with OS protocols tailored to the patient's biological characteristics.

Letrozole is a selective oral aromatase inhibitor that blocks estrogen synthesis by targeting the final step of its biosynthetic pathway. Data over the past two decades show letrozole's efficacy and safety in FP for hormone-positive breast cancer patients. A *meta*-analysis of 2,121 patients found that adding letrozole did not negatively impact the number of mature oocytes collected or other FP endpoints (Oktay et al., 2005, Bonardi et al., 2020). No significant rise in short-term breast cancer recurrence was observed in women using the letrozole protocol, even after multiple cycles (Azim et al., 2008). A study with 337 breast cancer patients, including 120 who underwent OS with letrozole, showed no significant increase in recurrence over five years (Kim et al., 2016). Another study using liquid biopsy found no increase in circulating tumor DNA in 14 out of 15 patients before/after OS with letrozole, supporting its safety (Rothé et al., 2021).

Last year's POSITIVE trial results provided insights on the effects of a temporary two-year interruption of adjuvant endocrine therapy for pregnancy attempts in women with hormone-sensitive breast cancer. Among 368 women who achieved pregnancy, 40 % used assisted reproductive technology. Over three years, there was no increase in breast cancer events in those who became pregnant compared to a control group (Partridge et al., 2023). This suggests that pregnancy does not negatively affect hormone-sensitive breast cancer relapse. Further evidence from a *meta*-analysis showed similar overall survival rates between women who did and did not become pregnant after early invasive breast cancer (Arecco et al., 2023).

Using letrozole for FP in breast cancer patients provides a foundation for its potential use in early-stage SBOT. Early-stage SBOT poses unique challenges, especially in preserving reproductive organs. Currently, data on the oncological safety of letrozole for FP in gynecological tumors is limited to case series.

Preliminary data from our case report suggests that OS with letrozole is feasible for FP in selected early-stage SBOT patients. Both patients successfully achieved OS and oocyte cryopreservation. Although Patient 2 experienced a recurrence, it is unclear if it was due to OS or pregnancy, as no recurrence signs were present during in-vitro fertilization treatment, and the first recurrence was diagnosed during C-section. The recurrence was confined to the remaining ovary, with no pelvic or peritoneal spread, and the oncological staging remained FIGO IC3. A second, slow-growing recurrence occurred, but its size remained stable during OS and increased slowly during pregnancy (from 8 to 11 mm). This approach allowed the young nulliparous patient to carry two pregnancies to term. Long-term monitoring and follow-up are essential to evaluate recurrence risk and reproductive outcomes in these patients.

## 4 Conclusion

In conclusion, this case report provides preliminary insights into the safety and efficacy of OS with letrozole in early-SBOT patients. The successful OS and oocyte cryopreservation, coupled with the absence of changes in the appearance of ovarian cysts during letrozole treatment, suggest that the use of letrozole did not have a negative impact on the disease course of SBOT in these 2 cases and underscore its potential for FP. While our findings are reassuring, they should be interpreted with caution and should not be extrapolated to other ovarian neoplasia which have a known hormonal sensitivity, such as low grade serous ovarian tumors. Future prospective studies with careful monitoring of both fertility outcomes and oncological safety are essential. As fertility-sparing surgery is now recommended for SBOT at any stage, ongoing research is crucial to ensure that FP strategies are both safe and effective (Rothé et al., 2021).

## 5 Disclosure

The authors have no relevant affiliations or financial involvement with any organization or entity with a financial interest in or financial conflict with the subject matter or materials discussed in the manuscript.

## CRediT authorship contribution statement

**Valeria Lombardi Fäh:** Writing – original draft. **Federico Del Vento:** Writing – review & editing, Data curation. **S. Intidhar Labidi-Galy:** Writing – review & editing, Visualization, Validation. **Manuela Undurraga:** Project administration.

## Informed consent

Written informed consent was obtained from the patient for publication of this case report and accompanying images. A copy of the written consent is available for review by the Editor-in-Chief of this journal on request.

## Declaration of competing interest

The authors declare that they have no known competing financial interests or personal relationships that could have appeared to influence the work reported in this paper.
